# Prediction of Functional Outcome in Stroke Patients with Proximal Middle Cerebral Artery Occlusions Using Machine Learning Models

**DOI:** 10.3390/jcm12030839

**Published:** 2023-01-20

**Authors:** Burak B. Ozkara, Mert Karabacak, Omar Hamam, Richard Wang, Apoorva Kotha, Neda Khalili, Meisam Hoseinyazdi, Melissa M. Chen, Max Wintermark, Vivek S. Yedavalli

**Affiliations:** 1 Department of Neuroradiology, MD Anderson Cancer Center, Houston, TX 77030, USA; 2 Department of Neurosurgery, Mount Sinai Health System, New York, NY 10029, USA; 3 Russell H. Morgan Department of Radiology and Radiological Sciences, Johns Hopkins Hospital, Baltimore, MD 21287, USA

**Keywords:** ischemic stroke, machine learning, medical decision making, middle cerebral artery, artificial intelligence

## Abstract

At present, clinicians are expected to manage a large volume of complex clinical, laboratory, and imaging data, necessitating sophisticated analytic approaches. Machine learning-based models can use this vast amount of data to create forecasting models. We aimed to predict short- and medium-term functional outcomes in acute ischemic stroke (AIS) patients with proximal middle cerebral artery (MCA) occlusions using machine learning models with clinical, laboratory, and quantitative imaging data as inputs. Included were consecutive AIS patients with MCA M1 and proximal M2 occlusions. The XGBoost, LightGBM, CatBoost, and Random Forest were used to predict the outcome. Minimum redundancy maximum relevancy was used for selecting features. The primary outcomes were the National Institutes of Health Stroke Scale (NIHSS) shift and the modified Rankin Score (mRS) at 90 days. The algorithm with the highest area under the receiver operating characteristic curve (AUROC) for predicting the favorable and unfavorable outcome groups at 90 days was LightGBM. Random Forest had the highest AUROC when predicting the favorable and unfavorable groups based on the NIHSS shift. Using clinical, laboratory, and imaging parameters in conjunction with machine learning, we accurately predicted the functional outcome of AIS patients with proximal MCA occlusions.

## 1. Introduction

With 12.2 million new cases yearly, acute ischemic stroke (AIS) is a significant cause of morbidity worldwide [1]. In approximately 30% of AIS patients, the underlying cause is large vessel occlusion. Furthermore, the most common artery involved in AIS is the middle cerebral artery (MCA) [2]. Due to the high incidence of large vessel MCA occlusions and treatment advancements in M1 and proximal M2 occlusions, outcome prognostication in patients affected by these specific occlusions has become increasingly significant [3,4,5,6].

Research has been conducted to identify methods for enhancing the precision of clinical predictions of short-term and mid-term outcomes following an AIS, which may be helpful in treatment decisions and handling the expectations of the patients and their families. Clinicians are increasingly expected to handle a greater volume of complex clinical, lab, and imaging data, resulting in the use of more sophisticated analytical approaches. This significant volume of clinical, lab, and imaging data can be utilized by machine learning (ML)-based prediction models to generate effective prognostication models. In the future, it might be possible to incorporate these models into clinical practice, where they can be utilized as decision-support aids. ML and deep learning models have been utilized in AIS research for tasks including diagnosis, radiological outcome prediction, morbidity and mortality prediction, and functional outcome prediction. Recently, Mainali et al. reviewed thirteen papers that predicted the functional outcome of AIS patients using ML or deep learning [7]. Adopting ML algorithms, according to them, allows us to efficiently process the vast amount of information that surrounds us [7].

According to our review of the literature, no study has investigated the ability of ML models to predict short-term and mid-term functional outcomes in AIS patients due to isolated MCA occlusions. As AIS due to MCA occlusions is approximately five times more prevalent than AIS due to isolated anterior and posterior cerebral artery occlusions, the development of ML models that predict outcomes in MCA occlusions is beneficial from a clinical perspective [8]. Functional outcomes in AIS patients with MCA occlusions were predicted by Forkert et al.; however, magnetic resonance imaging (MRI) scans were used as the input [9]. In this study, we aimed to predict short-term and mid-term functional outcomes in AIS patients due to proximal MCA occlusions with ML models using the clinical, lab, and quantitative imaging data as inputs.

## 2. Materials and Methods

### 2.1. The Patient Selection

In this retrospective cohort study, two comprehensive stroke centers including consecutive AIS patients admitted to Johns Hopkins Hospital (JHH) and Johns Hopkins Bayview Medical Center (JHBMC) between 1 October 2017 and 30 September 2022, were evaluated for eligibility. We searched for patients with MCA M1 and proximal M2 occlusions. The M1 segment of the MCA is defined as the horizontal portion of the proximal MCA from the bifurcation of the internal carotid artery (ICA) to the genu of the MCA branch or branches at the entrance to the insula [5]. M2 section was defined as vertical MCA branches within the Sylvian fissure that extend from the genu to the next genu at the level of the operculum [5]. Proximal M2 occlusions were defined as occlusions within 1 cm of the bifurcation of the MCA in the horizontal M2 section [10]. An anterior temporal artery arising from the horizontal M1 segment was not considered an M2 branch. AIS was diagnosed clinically and confirmed by brain computed tomography (CT). Patients who met the following inclusion criteria were included in the study: (1) Admitted within 24 h of symptom onset; (2) minimum 18 years old; (3) initial non-contrast brain CT scan excluded intracranial hemorrhage; (4) diagnosed with primary AIS due to M1 and proximal M2 occlusion based on CT angiography (CTA) and CT perfusion (CTP); (5) hypoperfusion analysis was performed utilizing an automated perfusion software platform based on CTP (RAPID 4.9, iSchemaView, Menlo Park, CA, USA); and (6) as the outcome measures, the National Institutes of Health Stroke Scale (NIHSS) shift or modified Rankin Score (mRS) at 90 days were available as the outcome measures. Patients with missing outcome data, secondary AIS due to emboli caused by endovascular treatment of another occlusion, and those discharged with a diagnosis of transient ischemic attack (TIA) were excluded. The Institutional Review Board at JHH approved the study. The study complied with the Health Insurance Portability and Accountability Act.

### 2.2. Data Extraction

From electronic medical records, demographic and clinical information were extracted retrospectively. The following variables were obtained: Sex, age, race, alcohol consumption, smoking status, whether JHH or JHBMC was the initial hospital, stroke etiology based on TOAST criteria [11], medical history (hypertension, diabetes mellitus, atrial fibrillation, heart disease, dyslipidemia, chronic kidney disease, deep venous thrombosis or pulmonary embolus at the time of admission, hepatitis C virus [HCV] and human immunodeficiency virus [HIV] status, sleep apnea, prior stroke or TIA, peripheral vascular disease, obesity, history of malignancy, anticoagulation or antiplatelet use), admission vitals (heart rate, systolic blood pressure, diastolic blood pressure, respiratory rate), admission shock index (SI), age-related admission SI, admission SpO_2_, admission body mass index (BMI), discharge vitals (heart rate, systolic blood pressure, diastolic blood pressure, respiratory rate), discharge BMI, discharge SpO_2_, admission NIHSS score, discharge NIHSS score, intravenous (IV) tissue plasminogen activator (tPA) treatment, mechanical thrombectomy (MT), and the mRS at 90 days following hospital discharge.

All patients had peripheral venous blood drawn in accordance with our local stroke care standard protocol at the emergency department. All blood samples were collected, processed, and analyzed using the same procedures. The following admission and discharge lab parameters were retrospectively extracted: Sodium, potassium, glucose, calcium, blood urea nitrogen (BUN), creatinine, hematocrit, hemoglobin, white blood cell (WBC) count, platelet count, and platelet count to WBC count ratio.

To collect radiologic variables, imaging reports were used. In addition, all CTAs were evaluated by a board-certified neuroradiologist (Vivek Srikar Yedavalli, 6 years of experience) in conjunction with all available imaging and clinical data for each patient in the study. The existence and precise location of any AIS were noted. The same neuroradiologist collected and confirmed the baseline Alberta Stroke Program Early CT Score (ASPECTS), the occluded vessel, the occluded segment, the occlusion laterality, the presence of hyperdense MCA on thin cuts, the presence of hemorrhagic transformation, and the type of hemorrhagic transformation. The time from admission to CT in minutes was extracted retrospectively. 

At the discretion of the neurointerventionalist, thrombectomy was performed by one of four experienced interventional neuroradiologists or endovascular neurosurgeons using any FDA-approved thrombectomy device. Extracted data included reperfusion grade as evaluated by the treating interventionalist after the procedure using the modified treatment in cerebral ischemia (mTICI) score, the number of passes during the thrombectomy procedure, time from admission to groin puncture in minutes, time from groin puncture to recanalization in minutes, time from admission to recanalization in minutes, and type of anesthesia during thrombectomy. If IV-tPA was administered, time from admission to needle time in minutes was extracted.

### 2.3. Imaging Protocols

Comprehensive CT imaging was performed at JHH and JHBMC from 1 October 2017 to 30 September 2022, utilizing helical scanners on the Siemens Flash and Drive systems (Siemens Healthineers, Erlangen, Germany). The parameters listed below are applicable to both Siemens scanners. Parameters for non-contrast CT: Helical mode at 5-mm slice thickness (ST), 120 kVp, 365 mAs, rotation time 1 s, acquisition time 6–8 s, collimation 128 × 0.6 mm, pitch value 0.55, scan direction CC. CTP parameters: Injection of 50 mL non-ionic iodinated contrast with 30 mL saline flush at 5–6 mL/s with 70–100 mm coverage at 5-mm ST. CTP parameters: 70 kVp, 200 mAs effective, rotation time 0.25 s, average acquisition time 60 s, collimation 48 × 1.2 mm, pitch value 0.70, 4D range 114 mm × 1.5 s. CTP images are then post-processed with commercial software RAPID 4.9 (IschemaView, Menlo Park, CA, USA) to generate Tmax maps. CTA head and neck parameters: Non-ionic iodinated contrast with 50–70 mL injected at 5–6 mL/s from the aortic arch to the vertex using a bolus-triggered injection technique at 3 mm ST. CTA parameters: 90/150 kVp, Sn filter, quality reference mAs 180, rotation time 0.25 s, average acquisition time 3–5 s, collimation 128 × 0.6 mm, pitch value 0.70, scan direction CC.

Extracted data included relative cerebral blood flow (rCBF) < 20%, rCBF < 30%, rCBF < 34%, rCBF < 38%, time to the maximum of the residue function (Tmax) > 4 s, Tmax > 6 s, Tmax > 8 s, Tmax > 10 s, cerebral blood volume (CBV) < 34%, CBV < 38%, CBV < 42%, mismatch volume, mismatch ratio, hypoperfusion index (HI), digital subtraction angiography (DSA) collateral score, clot burden score, and single-phase CTA collateral score [12]. The American Society of Interventional and Therapeutic Neuroradiology/Society of Interventional Radiology (ASITN/SIR) score was used for the collateral score for DSA. The mismatch volume was calculated by subtracting the perfusion deficit volume from the ischemic core volume. The mismatch ratio was calculated by dividing the perfusion deficit by the infarct core volume. HI is defined as the volumetric ratio of tissue with a Tmax > 10 s and Tmax > 6 s.

### 2.4. Outcome of Interest

NIHSS shift and mRS at 90 days were the primary outcomes. NIHSS shift (admission NIHSS score − discharge NIHSS score) was computed based on the recent study from Meyer et al. for each patient [13,14]. With a cut-off defined by the median NIHSS shift, patients with an NIHSS shift above the median score and patients with an NIHSS shift below the median score were assigned to the favorable outcome group and the unfavorable outcome group, respectively. Regarding mRS, the favorable outcome was defined as mRS 0 to 2 [15]. Our study aims to evaluate the effectiveness of ML algorithms in predicting favorable and unfavorable outcome groups based on mRS and NIHSS shift.

### 2.5. Data Preprocessing

Imputation was utilized to prevent the introduction of bias by removing missing data from patients. Values regarding MT related time-sensitive variables (time from admission to groin puncture, time from groin puncture to recanalization, time from admission to recanalization) for the patients who did not undergo MT, were assigned as the maximum value of the total patient cohort. Similarly, values regarding IV-tPA treatment related time-sensitive variables (time from admission to needle time) were assigned as the maximum value of the total patient cohort. After this manual imputation for the non-applicable variables, missing values for the remaining continuous variables were imputed with the nearest neighbor (NN) method after removing variables with missing values for more than 25% of the patient cohort [16]. 

The robust scaler was used for continuous data to adjust outliers [17]. The Min-Max normalization method was applied to normalize the data, and each continuous variable was set between the 0 and 1 range [18]. Ordinal categorical variables (such as mTICI) were coded with the ordinal encoder [19], while non-binary variables (e.g., race, sex) were one-hot-encoded [20]. Minimum redundancy maximum relevance feature selection approach was utilized for feature selection [21]. Since the NIHSS shift is an outcome obtained at the time of discharge, other parameters obtained at the time of discharge were not used to predict the groups stratified based on the NIHSS shift. Parameters obtained at discharge were used to predict mRS at 90 days.

### 2.6. Modeling, Training, Validation, and Test Sets

We divided our sample at a ratio of 60:20:20. Therefore, 60% of the data were allocated to the training set, 20% to the validation set, and the remaining 20% to the test set. The training set was used to build the models, the validation set to fine-tune the hyperparameters, and the test set to evaluate the models’ performance.

Python 3.7.15 was used to run ML analyses. We used four supervised ML algorithms: CatBoost, XGBoost, LightGBM, and Random Forest. With the Optuna optimization package, the area under the receiver operating characteristic curve was optimized (AUROC). The Optuna streamlines the utilization of various cutting-edge optimization methods for efficient and rapid hyperparameter optimization [22]. The Bayesian optimization algorithm Tree-Structured Parzen Estimator Sampler (TPESampler) was used to create AUROC estimations that served as a guide for the optimization phase. The final models were formed using the training set and optimized hyperparameters.

### 2.7. Performance Evaluation

Visually, algorithms were assessed using the receiver operating characteristic (ROC) curve and the precision-recall curve (PRC); and numerically, using the AUROC, the area under the PRC (AUPRC), accuracy, Matthew’s correlation coefficient (MCC), recall, and precision. In addition to performance charts and metrics, we utilized SHapley Additive exPlanations (SHAP) to assess the relative significance of predictor factors. SHAP is a method for illustrating how ML algorithms yield predictions.

### 2.8. Statistical Analysis

All statistical analyses were performed in Python version 3.7.15. The descriptive analyses were shown as means (± standard deviations) for normally distributed continuous variables, medians (interquartile ranges) for non-normally distributed continuous variables, and the number of patients (% percentages). The independent t-test for normally distributed continuous variables with equal variances and the Welch’s t-test for normally distributed continuous variables with unequal variances were used to assess group differences in outcomes. Group differences in outcomes were investigated using the Mann-Whitney U test for non-normally distributed continuous variables and Pearson’s chi-squared test for categorical variables. Levene’s test was used to evaluate the equality of variances for a variable, whereas the Shapiro-Wilk test was utilized to assess normality. The differences were considered statistically significant at a *p*-value of less than 0.05.

## 3. Results

Initially, 279 patients admitted to JHH and JHBMC with AIS due to an M1 or proximal M2 occlusion or both were included in the study. Forty-nine patients were excluded due to unavailable outcome data. Additionally, 45 patients had only the NIHSS shift, 42 patients had only the mRS, and 143 patients had both the NIHSS shift and the mRS available. Therefore, our study included a total of 230 patients, 185 of whom were included in predicting favorable and unfavorable outcomes based on mRS and 188 based on NIHSS shift. Using the minimum redundancy maximum relevance feature selection approach, each model used 20 features as input.

### 3.1. mRS as the Outcome Measure

**Figure 1:**
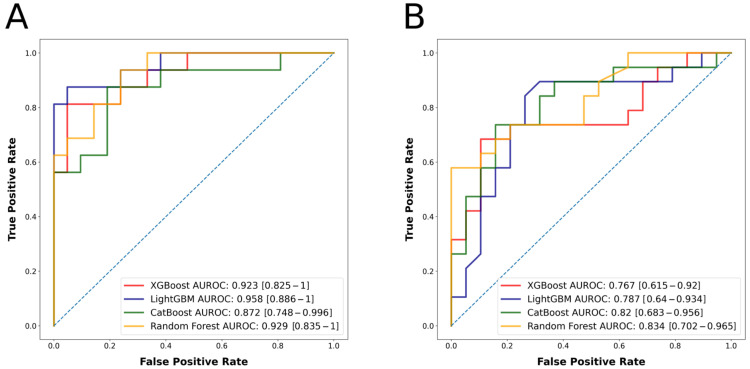
(**A**) Algorithms’ receiver operating characteristic curves for the outcome mRS at 90 days. (**B**) Algorithms’ receiver operating characteristic curves for the outcome NIHSS shift.

**Figure 2:**
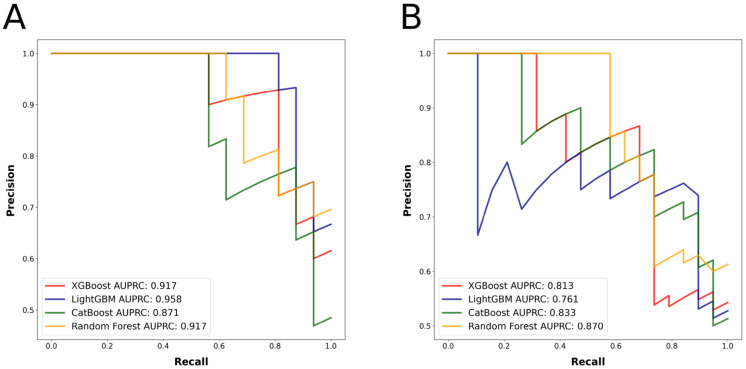
(**A**) Algorithms’ precision-recall curves for the outcome mRS at 90 days. (**B**) Algorithms’ precision-recall curves for the outcome NIHSS shift.

**Figure 3:**
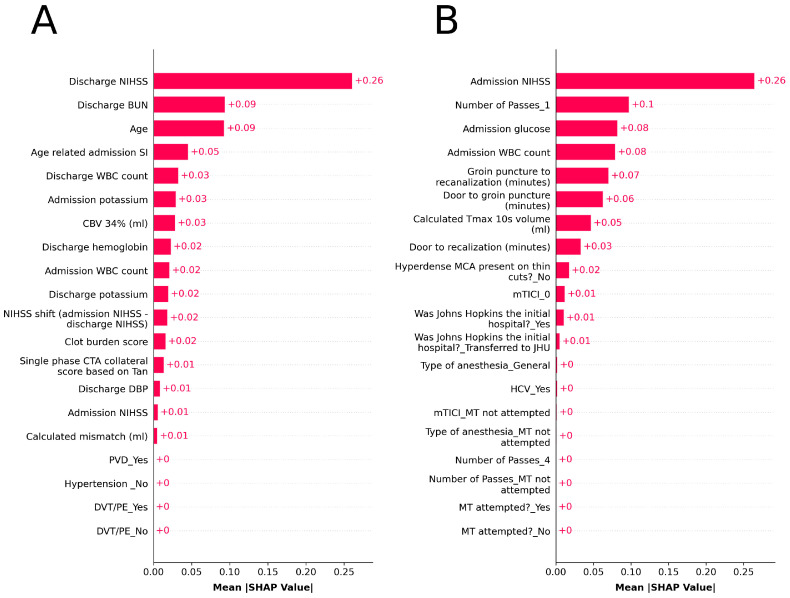
Sorted feature importance and their mean SHAP values (**A**) for the model predicting the mRS at 90 days with the LightGBM algorithm, (**B**) for the model predicting the NIHSS shift with the Random Forest algorithm.

There were 99 patients in the group with a favorable outcome (mRS 0–2) and 86 patients in the group with an unfavorable outcome (mRS 3–6). Table 1 displays the characteristics of the patient population, both by group and in total. The best predicting algorithm in terms of AUROC was LightGBM, with an AUROC of 0.958 (confidence interval [CI] = 0.886–1). Once again, LightGBM had the highest AUPRC value of 0.958. Table 2 provides detailed metrics regarding the performance of the algorithms. Figure 1A depicts the ROC curve, while Figure 2A depicts the PRC. After feature selection, the models utilized the 20 most significant features for outcome prediction. Figure 3A displays SHAP plot of the LightGBM algorithm with 20 selected features. SHAP plots of other algorithms predicting mRS at 90 days can be found in Supplementary Figure S1.

### 3.2. NIHSS Shift as the Outcome Measure

There were 89 patients in the group with a favorable outcome and 99 patients in the group with an unfavorable outcome. The characteristics of the patient population, both by group and overall, are presented in Table 3. The best predicting algorithm in terms of AUROC was Random Forest, with an AUROC of 0.834 (CI = 0.702–0.965). Once again, Random Forest algorithm had the highest AUPRC value of 0.870. Table 2 provides comprehensive performance metrics for the models. Figure 1B displays the ROC curve, while 2B shows the PRC curve. After feature selection, models used the 20 most significant features to predict the outcome. Figure 3B depicts SHAP plot of the Random Forest algorithm with 20 selected features. SHAP plots of other algorithms predicting the NIHSS shift can be found in Supplementary Figure S2.

## 4. Discussion

To the best of our knowledge, this is the first study to predict functional outcomes in AIS patients with isolated proximal MCA occlusions. This study presents a series of ML models that accurately predict the groups stratified based on NIHSS shift (short-term functional outcome) and mRS at 90 days (mid-term functional outcome) in AIS patients with isolated proximal MCA occlusions. Managing massive and varied data, detecting subtle and hidden patterns, and forecasting complicated events are the primary benefits of ML models. ML models have the potential to enhance clinical management by providing patient-centered, individualized management based on the outcomes of the model. Physicians may be able to personalize better patient care plans for those at risk of unfavorable outcomes, and they can provide patients with more accurate information regarding the prognosis of their functional status. On the other hand, physicians may direct ML models by identifying crucial features to include and the appropriate sources to represent those features. This study adds to the body of knowledge by describing the advantages and efficacy of incorporating ML into patient care to predict outcomes in AIS patients [7]. The most successful algorithm was LightGBM in predicting the favorable and unfavorable outcome groups based on mRS at 90 days with an AUROC of 0.958 (CI = 0.886–1) and an AUPRC of 0.958. The algorithm with the highest AUROC and AUPRC was Random Forest, with 0.834 (CI = 0.702–0.965) and 0.870, respectively, when predicting the favorable and unfavorable groups based on the NIHSS shift.

In the literature, ML and deep learning models have been used to predict favorable (mRS at 90 days 0–2) and unfavorable (mRS at 90 days 3–6) functional outcome groups in AIS patients. Despite the difficulties of directly comparing the performance of this algorithm with the performance of algorithms in other recent studies, based on the metrics reported, our model appeared to achieve similar performance to the best-performing models in other published studies [23,24,25]. Heo et al. used a deep learning model, which yielded an AUROC of 0.888; Monteiro et al. used an ML model, which produced an AUROC of 0.936; and Brugnara et al. used an ML model, which yielded an AUROC of 0.856 [23,24,25]. Our algorithm LightGBM predicted the mRS group with an AUROC of 0.958, outperforming all other models. Notably, while our model included only 20 features, the model of Monteiro et al., which was the most comparable to ours in terms of the AUROC, contained 152 features. Their model with 49 features yielded an AUROC of 0.808. Furthermore, Brugnara et al. reported an accuracy of 0.804 for their ML model, whereas our model achieved an accuracy of 0.892 [25]. Moreover, Brugnara et al. had 246 patients, Monteiro et al. had 399 patients, and Heo et al. had 2604 patients in their best-performing models, whereas we had 185 patients. Although the number of patients in our study was relatively small, we were able to achieve similar results compared to these papers, demonstrating the efficacy of our models. While it is not accurate to directly compare models based on AUROC value, we utilized this metric to compare our model to theirs since they did not report AUPRC and accuracy.

In our study, we also used SHAP to create visual explanations for the predictions of the four different algorithms used in this study. LightGBM was the most accurate model for predicting mRS at 90 days, with discharge NIHSS score, discharge BUN, age, age-related admission SI, and discharge WBC count being the top five most important features according to SHAP results. Brugnara et al. reported their top five features with the greatest importance for predicting mRS, and they were NIHSS score after 24 h, premorbid mRS score, final infarction volume on post-interventional CT after 18 to 36 h, interval from groin puncture to recanalization, and baseline acute ischemic volume on native CT [25]. With the exception of the time between groin puncture and recanalization, we did not utilize four of the top five factors in their analysis. Therefore, comparing our top parameters to theirs is challenging. In their best-performing models, Heo et al. used 38 features, while Monteiro et al. used 152 features, with no SHAP figures or other metrics indicating the importance of distinctive features in predicting the outcome [23,24]. Furthermore, our SHAP results reveal that patient discharge metrics are crucial for predicting the mRS at 90 days. While reliable scores that can predict the patient’s functional outcomes use available metrics at admission, our results indicate that utilization of discharge parameters might increase the accuracy of the prediction [26]. This can be an open area of research.

No prior study has, to our knowledge, utilized ML approaches to analyze the NIHSS shift. Therefore, we cannot compare the performance of our predictive models for the NIHSS shift to what was accomplished before. The SHAP analysis of Random Forest, the best predicting algorithm in terms of AUROC and AUPRC, for the NIHSS shift revealed that the most influential variables were admission NIHSS score, number of passes in thrombectomy, admission glucose, admission WBC count, and groin puncture to recanalization in minutes. As it is well known that stroke severity is a crucial predictor of outcomes in AIS patients, it is not surprising that admission NIHSS was the most important variable [27]. The SHAP analysis revealed that thrombectomy-related parameters were crucial, demonstrating the significance of treatment, which makes intuitive sense given that the NIHSS shift occurs during hospitalization. Furthermore, glucose levels at admission were identified as a crucial parameter, which makes sense given that elevated glucose levels are independent predictors of poor outcome in stroke patients [28]. Furthermore, admission WBC count was identified as a significant predictor of the outcome, consistent with earlier research [29].

Despite the rigor of our methods, our study is not without limitations. Our study has limitations inherent to retrospective studies. To validate our ML models, prospective studies with a larger sample size would be needed. We could not utilize periprocedural vital signs, such as blood pressure since they were not collected. Although the algorithms used in our study are widely regarded as state-of-the-art, it is worth noting that algorithms utilizing gradient boosting are susceptible to outliers and prone to overfitting. The robust scaler was used to handle outliers, and the minimum redundancy maximum relevance feature selection approach was used to prevent overfitting.

## 5. Conclusions

Using clinical, laboratory, and imaging parameters in conjunction with ML, the functional outcome of AIS patients with proximal MCA occlusions could be accurately predicted. These algorithms have the potential to be implemented in the decision-making process in clinical practice. This can potentially improve prognostic stratification and management.

## Figures and Tables

**Table 1:** The characteristics of the patient population utilized in mRS prediction.

Variables		90-Day mRS (0–2)	90-Day mRS (3–6)	*p* Values	Total
		Mean (± SD), Median (IQR), or *n* (%)	Mean (± SD), Median (IQR), or *n* (%)		Mean (± SD), Median (IQR), or *n* (%)
Age		62.23 (± 15.81)	76.0 (16.5)	<0.001	69.0 (22.0)
Sex	Female	57 (57.6%)	54 (62.8%)	0.568	111 (60.0%)
	Male	42 (42.4%)	32 (37.2%)		74 (40.0%)
Race	White	60 (60.6%)	41 (47.7%)	0.206	101 (54.6%)
	Black/African American	34 (34.3%)	40 (46.5%)		74 (40.0%)
	Other	5 (5.0%)	5 (5.8%)		10 (5.4%)
Alcohol	Not current alcohol user	63 (63.6%)	70 (81.4%)	0.012	133 (71.9%)
	Current alcohol user	36 (36.4%)	16 (18.6%)		52 (28.1%)
Smoking	Never smoker	57 (57.6%)	49 (57.0%)	1.0	106 (57.3%)
	Current or former smoker	42 (42.4%)	37 (43.0%)		79 (42.7%)
Was Johns Hopkins the initial hospital?	No, ttransferred to JHH	26 (26.3%)	19 (22.1%)	0.626	45 (24.3%)
	Yes	73 (73.7%)	67 (77.9%)		140 (75.7%)
Hypertension	No	31 (31.3%)	13 (15.1%)	0.016	44 (23.8%)
	Yes	68 (68.7%)	73 (84.9%)		141 (76.2%)
Diabetes	No	77 (77.8%)	52 (60.5%)	0.017	129 (69.7%)
	Yes	22 (22.2%)	34 (39.5%)		56 (30.3%)
Atrial fibrillation	No	63 (63.6%)	54 (62.8%)	1.0	117 (63.2%)
	Yes	36 (36.4%)	32 (37.2%)		68 (36.8%)
Heart disease	No	56 (56.6%)	42 (48.8%)	0.367	98 (53.0%)
	Yes	43 (43.4%)	44 (51.2%)		87 (47.0%)
Dyslipidemia	No	52 (52.5%)	42 (48.8%)	0.724	94 (50.8%)
	Yes	47 (47.5%)	44 (51.2%)		91 (49.2%)
Chronic kidney disease	No	84 (84.8%)	67 (77.9%)	0.305	151 (81.6%)
	Yes	15 (15.2%)	19 (22.1%)		34 (18.4%)
Deep vein thrombosis/Pulmonary embolism	No	94 (95.0%)	66 (76.7%)	0.001	160 (86.5%)
	Yes	5 (5.0%)	20 (23.3%)		25 (13.5%)
Hepatitis C virus	No	95 (96.0%)	84 (97.7%)	0.81	179 (96.8%)
	Yes	4 (4.0%)	2 (2.3%)		6 (3.2%)
Human immunodeficiency virus	No	98 (99.0%)	84 (97.7%)	0.902	182 (98.4%)
	Yes	1 (1.0%)	2 (2.3%)		3 (1.6%)
Sleep apnea	No	88 (88.9%)	78 (90.7%)	0.872	166 (89.7%)
	Yes	11 (11.1%)	8 (9.3%)		19 (10.3%)
Prior stroke/TIA	No	80 (80.8%)	69 (80.2%)	1.0	149 (80.5%)
	Yes	19 (19.2%)	17 (19.8%)		36 (19.5%)
Peripheral vascular disease	No	96 (97.0%)	72 (83.7%)	0.004	168 (90.8%)
	Yes	3 (3.0%)	14 (16.3%)		17 (9.2%)
Obesity	No	67 (67.7%)	60 (69.8%)	0.883	127 (68.6%)
	Yes	32 (32.3%)	26 (30.2%)		58 (31.4%)
History of malignancy	No	83 (83.8%)	70 (81.4%)	0.808	153 (82.7%)
	Yes	16 (16.2%)	16 (18.6%)		32 (17.3%)
Antiplatelet or anticoagulation use	No	65 (65.7%)	49 (57.0%)	0.289	114 (61.6%)
	Yes	34 (34.3%)	37 (43.0%)		71 (38.4%)
Admission BMI		27.82 (9.3)	27.49 (6.36)	0.138	27.54 (6.99)
Admission HR		82.0 (25.0)	84.0 (25.5)	0.184	82.0 (26.0)
Admission SBP		143.0 (33.0)	154.06 (± 28.8)	0.132	146.0 (34.0)
Admission DBP		86.0 (24.5)	83.0 (25.75)	0.327	84.0 (25.0)
Admission RR		17.0 (2.0)	18.0 (4.0)	0.133	18.0 (3.0)
Admission SpO_2_		98.0 (3.0)	98.5 (3.0)	0.307	98.0 (3.0)
Admission SI		0.59 (0.22)	0.59 (± 0.15)	0.932	0.58 (0.21)
Age related admission SI		34.07 (13.47)	43.08 (± 14.55)	<0.001	37.89 (17.03)
Admission sodium		138.7 (± 3.21)	139.0 (4.75)	0.818	139.0 (4.0)
Admission potassium		4.1 (± 0.45)	4.15 (0.5)	0.368	4.1 (0.6)
Admission glucose		117.0 (36.0)	121.0 (47.75)	0.176	118.0 (38.0)
Admission calcium		8.98 (± 0.57)	9.03 (± 0.61)	0.58	9.0 (± 0.59)
Admission BUN		15.0 (8.0)	19.0 (9.75)	0.001	16.0 (8.0)
Admission creatinine		0.9 (0.3)	1.0 (0.51)	0.054	0.96 (0.4)
Admission hematocrit		39.46 (± 4.74)	40.25 (± 6.03)	0.319	39.83 (± 5.38)
Admission hemoglobin		12.84 (± 1.71)	13.0 (± 2.15)	0.578	12.91 (± 1.92)
Admission WBC count		7.48 (3.23)	8.32 (4.4)	0.048	7.96 (3.79)
Admission platelet count (×1000)		227.0 (105.0)	214.8 (107.75)	0.288	220.0 (107.0)
Admission platelet count (×1000) to WBC count ratio		30.28 (13.14)	25.79 (12.75)	0.002	28.88 (11.78)
Admission NIHSS		12.0 (11.0)	17.12 (± 7.07)	<0.001	14.0 (10.0)
Door to CT (minutes)		23.0 (27.0)	25.5 (28.75)	0.334	25.0 (30.0)
Baseline non-contrast CT ASPECTS on 5-mm cuts		9.0 (2.0)	9.0 (4.0)	0.013	9.0 (2.8)
Hyperdense MCA present on thin cuts?	No	42 (42.4%)	36 (41.9%)	1.0	78 (42.2%)
	Yes	57 (57.6%)	50 (58.1%)		107 (57.8%)
Stroke etiology (TOAST criteria)	Cardioembolism	49 (49.5%)	45 (52.3%)	0.873	94 (50.8%)
	Large artery atherosclerosis	17 (17.2%)	17 (19.8%)		34 (18.4%)
	Stroke of undetermined etiology	27 (27.3%)	20 (23.3%)		47 (25.4%)
	Stroke of other determined etiology	6 (6.1%)	4 (4.6%)		10 (5.4%)
Laterality	Left	44 (44.4%)	47 (54.6%)	0.27	91 (49.2%)
	Right	54 (54.6%)	39 (45.4%)		93 (50.3%)
	Bilateral	1 (1.0%)	0 (0.0%)		1 (0.5%)
Occluded segment	M1	51 (51.5%)	43 (50.0%)	0.879	94 (50.8%)
	M2	20 (20.2%)	20 (23.3%)		40 (21.6%)
	M1 and M2	28 (28.3%)	23 (26.7%)		51 (27.6%)
Hemorrhagic transformation?	No	72 (72.7%)	50 (58.1%)	0.053	122 (66.0%)
	Yes	27 (27.3%)	36 (41.9%)		63 (34.0%)
Type of hemorrhagic transformation	No HT	72 (72.7%)	50 (58.1%)	0.058	122 (66.0%)
	HI1	3 (3.0%)	2 (2.3%)		5 (2.7%)
	HI2	15 (15.2%)	13 (15.1%)		28 (15.1%)
	PH1	8 (8.1%)	15 (17.4%)		23 (12.4%)
	PH2	1 (1.0%)	6 (7.0%)		7 (3.8%)
Calculated rCBF 20% volume (mL)		0.0 (6.9)	0.0 (13.7)	0.186	0.0 (8.0)
Calculated rCBF 30% volume (mL)		8.0 (23.0)	9.8 (33.45)	0.226	8.0 (26.6)
Calculated rCBF 34% volume (mL)		12.0 (31.2)	15.0 (36.45)	0.166	14.8 (33.4)
Calculated rCBF 38% volume (mL)		20.0 (33.0)	22.0 (43.15)	0.127	21.4 (35.0)
Calculated Tmax 4 s volume (mL)		190.0 (108.3)	216.0 (139.25)	0.03	205.0 (136.8)
Calculated Tmax 6 s volume (mL)		101.0 (81.0)	116.1 (83.75)	0.013	106.2 (80.0)
Calculated Tmax 8 s volume (mL)		54.0 (64.0)	74.0 (67.5)	0.018	67.0 (63.2)
Calculated Tmax 10 s volume (mL)		37.0 (55.0)	45.5 (62.55)	0.024	41.0 (54.0)
CBV 34% (mL)		0.0 (13.4)	5.0 (19.9)	0.031	3.0 (16.4)
CBV 38% (mL)		4.0 (16.0)	7.0 (23.25)	0.024	5.0 (19.2)
CBV 42% (mL)		4.2 (17.8)	8.5 (26.0)	0.015	7.0 (21.6)
Calculated mismatch (mL)		82.0 (67.6)	90.0 (70.75)	0.045	85.0 (68.6)
Calculated mismatch ratio		19.4 (35.06)	17.05 (36.55)	0.483	18.18 (35.5)
Hypoperfusion index		0.3 (0.3)	0.4 (0.3)	0.178	0.38 (0.3)
Single phase CTA collateral score based on Tan		2.0 (0.2)	1.6 (1.0)	0.003	2.0 (1.0)
DSA collaterals (ASITN/SIR criteria)		2.0 (1.0)	2.0 (2.0)	0.156	2.0 (2.0)
Clot burden score		6.8 (2.3)	6.0 (3.95)	0.026	6.0 (3.2)
IV TPA administered?	No	59 (59.6%)	67 (77.9%)	0.012	126 (68.1%)
	Yes	40 (40.4%)	19 (22.1%)		59 (31.9%)
Door to needle time (minutes)		1113.0 (1052.0)	1113.0 (0.0)	0.011	1113.0 (1036.0)
MT attempted?	No	23 (23.2%)	18 (20.9%)	0.843	41 (22.2%)
	Yes	76 (76.8%)	68 (79.1%)		144 (77.8%)
Type of anesthesia	General	68 (68.7%)	64 (74.4%)	0.561	132 (71.4%)
	Monitored anesthesia care	8 (8.1%)	4 (4.6%)		12 (6.5%)
	MT not attempted	23 (23.2%)	18 (20.9%)		41 (22.2%)
Door to groin puncture (minutes)		160.0 (463.2)	181.5 (574.75)	0.367	170.0 (595.4)
Groin puncture to recanalization (minutes)		33.0 (79.5)	46.4 (81.5)	0.183	38.0 (82.4)
Door to recanalization (minutes)		193.0 (469.0)	224.5 (552.5)	0.132	209.0 (602.0)
Number of Passes	1	62 (62.6%)	39 (45.4%)	0.027	101 (54.6%)
	2	10 (10.1%)	13 (15.1%)		23 (12.4%)
	3	1 (1.0%)	7 (8.1%)		8 (4.3%)
	4	1 (1.0%)	6 (7.0%)		7 (3.8%)
	5	1 (1.0%)	2 (2.3%)		3 (1.6%)
	6	0 (0.0%)	1 (1.2%)		1 (0.5%)
	7	1 (1.0%)	0 (0.0%)		1 (0.5%)
	MT not attempted	23 (23.2%)	18 (20.9%)		41 (22.2%)
mTICI	0	2 (2.0%)	6 (7.0%)	0.21	8 (4.3%)
	1	1 (1.0%)	3 (3.5%)		4 (2.2%)
	2a	1 (1.0%)	4 (4.6%)		5 (2.7%)
	2b	18 (18.2%)	15 (17.4%)		33 (17.8%)
	2c	8 (8.1%)	10 (11.6%)		18 (9.7%)
	3	46 (46.5%)	30 (34.9%)		76 (41.1%)
	MT not attempted	23 (23.2%)	18 (20.9%)		41 (22.2%)
Discharge sodium		139.0 (3.0)	140.0 (5.0)	0.077	140.0 (4.0)
Discharge potassium		4.13 (± 0.39)	4.3 (0.6)	0.004	4.2 (0.54)
Discharge glucose		104.0 (24.0)	113.5 (45.5)	0.105	107.0 (36.0)
Discharge calcium		8.8 (0.7)	8.8 (1.0)	0.062	8.8 (0.7)
Discharge BUN		14.0 (7.0)	19.0 (11.75)	<0.001	16.0 (10.0)
Discharge creatinine		0.85 (0.36)	0.9 (0.5)	0.176	0.9 (0.4)
Discharge hematocrit		36.33 (± 5.4)	33.22 (± 6.63)	0.001	34.89 (± 6.19)
Discharge hemoglobin		11.77 (± 1.91)	10.62 (± 2.33)	<0.001	11.24 (± 2.18)
Discharge WBC count		7.73 (2.54)	9.98 (4.19)	<0.001	8.42 (3.35)
Discharge platelet count (×1000)		223.0 (104.5)	258.6 (170.0)	0.097	239.0 (134.0)
Discharge platelet count (×1000) to WBC count ratio		30.38 (15.82)	26.24 (12.96)	0.043	28.87 (14.12)
Discharge BMI		26.97 (9.79)	27.41 (7.75)	0.583	27.14 (9.15)
Discharge SBP		126.0 (23.0)	128.3 (25.5)	0.916	127.0 (23.0)
Discharge DBP		74.3 (± 13.03)	67.9 (18.75)	0.013	71.0 (18.0)
Discharge HR		78.59 (± 15.23)	76.5 (22.85)	0.875	77.0 (23.0)
Discharge RR		18.0 (2.0)	18.0 (4.0)	0.186	18.0 (2.6)
Discharge SpO_2_		97.0 (3.0)	97.0 (4.0)	0.434	97.0 (3.0)
Discharge NIHSS		2.0 (3.0)	9.0 (8.0)	<0.001	4.0 (7.2)
NIHSS shift		9.0 (9.0)	4.96 (± 5.97)	<0.001	7.07 (± 6.39)
90-day mRS ^a^		1.0 (2.0)	5.0 (2.0)	<0.001	2.0 (4.0)

^a^ Not included as an input in the model. Abbreviations: mRS: Modified Rankin Score; SD: Standard deviation; IQR: Interquartile range; JHU: Johns Hopkins Hospitals; TIA: Transient ischemic attack; BMI: Body mass index; HR: Heart rate; SBP: Systolic blood pressure; DBP: Diastolic blood pressure; RR: Respiratory rate; SI: Shock index; BUN: Blood urea nitrogen; WBC: White blood cell; NIHSS: National Institutes of Health Stroke Scale; CT: Computed tomography; ASPECTS: Alberta Stroke Program Early CT Score; MCA: Middle cerebral artery; HI: Hemorrhagic infarction; PH: Parenchymal hematoma; rCBF: Relative cerebral blood flow; Tmax: Time to the maximum of the residue function; CBV: Cerebral blood volume; CTA: Computed tomography angiography; DSA: Digital subtraction angiography; ASITN/SIR: The American Society of Interventional and Therapeutic Neuroradiology/Society of Interventional Radiology; IV: Intravenous; tPA: Tissue plasminogen activator; MT: Mechanical thrombectomy; mTICI: Modified treatment in cerebral ischemia.

**Table 2:** Performance metrics of the algorithms.

	Algorithm	Precision	Recall	F1	Accuracy	MCC	AUROC (95% CI)	AUPRC
**mRS**	XGBoost	0.75	0.923	0.828	0.865	0.729	0.923 (0.825–1)	0.917
	LightGBM	0.812	0.929	0.867	0.892	0.781	0.958 (0.886–1)	0.958
	CatBoost	0.562	0.9	0.692	0.784	0.574	0.872 (0.748–0.996)	0.871
	Random Forest	0.625	0.909	0.741	0.811	0.626	0.929 (0.835–1)	0.917
	Mean	0.687	0.915	0.782	0.838	0.678	0.921	0.916
**NIHSS Shift**	XGBoost	0.737	0.7	0.718	0.711	0.422	0.767 (0.615–0.92)	0.813
	LightGBM	0.895	0.63	0.739	0.684	0.406	0.787 (0.64–0.934)	0.761
	CatBoost	0.737	0.737	0.737	0.737	0.474	0.820 (0.683–0.956)	0.833
	Random Forest	0.684	0.765	0.722	0.737	0.476	0.834 (0.702–0.965)	0.870
	Mean	0.763	0.708	0.729	0.717	0.445	0.802	0.819

Abbreviations: MCC: Matthew’s correlation coefficient; AUROC: Area under the receiver operating characteristic curve; CI: Confidence interval; AUPRC: The area under the precision-recall curve; mRS: Modified Rankin Score; NIHSS: National Institutes of Health Stroke Scale.

**Table 3:** The characteristics of the patient population utilized in NIHSS shift prediction.

Variables		High NIHSS Shift	Low NIHSS Shift	*p* Values	Total
		Mean (± SD), Median (IQR), or *n* (%)	Mean (± SD), Median (IQR), or *n* (%)		Mean (± SD), Median (IQR), or *n* (%)
Age		66.0 (17.0)	66.27 (± 15.16)	0.897	67.0 (19.25)
Sex	Female	53 (59.6%)	55 (55.6%)	0.685	108 (57.4%)
	Male	36 (40.4%)	44 (44.4%)		80 (42.6%)
Race	White	43 (48.3%)	48 (48.5%)	0.985	91 (48.4%)
	Black/African American	41 (46.1%)	46 (46.5%)		87 (46.3%)
	Other	5 (5.6%)	5 (5.0%)		10 (5.3%)
Alcohol	Not current alcohol user	64 (71.9%)	67 (67.7%)	0.637	131 (69.7%)
	Current alcohol user	25 (28.1%)	32 (32.3%)		57 (30.3%)
Smoking	Never smoker	57 (64.0%)	45 (45.4%)	0.016	102 (54.3%)
	Current or former smoker	32 (36.0%)	54 (54.6%)		86 (45.7%)
Was Johns Hopkins the initial hospital?	No, transferred to JHH	16 (18.0%)	32 (32.3%)	0.037	48 (25.5%)
	Yes	73 (82.0%)	67 (67.7%)		140 (74.5%)
Hypertension	No	17 (19.1%)	25 (25.2%)	0.403	42 (22.3%)
	Yes	72 (80.9%)	74 (74.8%)		146 (77.7%)
Diabetes	No	69 (77.5%)	68 (68.7%)	0.231	137 (72.9%)
	Yes	20 (22.5%)	31 (31.3%)		51 (27.1%)
Atrial fibrillation	No	53 (59.6%)	66 (66.7%)	0.39	119 (63.3%)
	Yes	36 (40.4%)	33 (33.3%)		69 (36.7%)
Heart disease	No	40 (44.9%)	58 (58.6%)	0.085	98 (52.1%)
	Yes	49 (55.1%)	41 (41.4%)		90 (47.9%)
Dyslipidemia	No	51 (57.3%)	48 (48.5%)	0.288	99 (52.7%)
	Yes	38 (42.7%)	51 (51.5%)		89 (47.3%)
Chronic kidney disease	No	70 (78.6%)	81 (81.8%)	0.718	151 (80.3%)
	Yes	19 (21.4%)	18 (18.2%)		37 (19.7%)
Deep vein thrombosis/Pulmonary embolism	No	78 (87.6%)	88 (88.9%)	0.969	166 (88.3%)
	Yes	11 (12.4%)	11 (11.1%)		22 (11.7%)
Hepatitis C virus	No	84 (94.4%)	95 (96.0%)	0.87	179 (95.2%)
	Yes	5 (5.6%)	4 (4.0%)		9 (4.8%)
Human immunodeficiency virus	No	88 (98.9%)	96 (97.0%)	0.69	184 (97.9%)
	Yes	1 (1.1%)	3 (3.0%)		4 (2.1%)
Sleep apnea	No	82 (92.1%)	87 (87.9%)	0.469	169 (89.9%)
	Yes	7 (7.9%)	12 (12.1%)		19 (10.1%)
Prior stroke/TIA	No	66 (74.2%)	81 (81.8%)	0.274	147 (78.2%)
	Yes	23 (25.8%)	18 (18.2%)		41 (21.8%)
Peripheral vascular disease	No	85 (95.5%)	91 (91.9%)	0.48	176 (93.6%)
	Yes	4 (4.5%)	8 (8.1%)		12 (6.4%)
Obesity	No	61 (68.5%)	63 (63.6%)	0.579	124 (66.0%)
	Yes	28 (31.5%)	36 (36.4%)		64 (34.0%)
History of malignancy	No	75 (84.3%)	79 (79.8%)	0.545	154 (81.9%)
	Yes	14 (15.7%)	20 (20.2%)		34 (18.1%)
Antiplatelet or anticoagulation use	No	48 (53.9%)	65 (65.7%)	0.136	113 (60.1%)
	Yes	41 (46.1%)	34 (34.3%)		75 (39.9%)
Admission BMI		27.37 (7.11)	29.01 (9.08)	0.293	28.47 (7.53)
Admission HR		82.0 (25.0)	82.0 (25.5)	0.519	82.0 (26.0)
Admission SBP		150.47 (± 25.54)	147.0 (43.5)	0.57	147.0 (32.25)
Admission DBP		81.0 (22.0)	88.75 (± 19.67)	0.199	85.0 (27.0)
Admission RR		18.0 (4.0)	18.0 (4.0)	0.112	18.0 (4.0)
Admission SpO_2_		98.0 (3.0)	98.0 (2.0)	0.441	98.0 (3.0)
Admission SI		0.56 (0.21)	0.56 (0.18)	0.989	0.56 (0.18)
Age related admission SI		36.4 (13.82)	35.38 (15.38)	0.913	36.12 (14.92)
Admission sodium		138.87 (± 3.4)	139.02 (± 3.39)	0.77	139.0 (4.0)
Admission potassium		4.12 (± 0.4)	4.1 (0.55)	0.935	4.1 (0.6)
Admission glucose		116.0 (40.0)	114.0 (36.0)	0.558	116.0 (38.0)
Admission calcium		8.92 (± 0.58)	9.1 (0.8)	0.058	9.0 (0.73)
Admission BUN		16.0 (8.0)	16.0 (7.0)	0.726	16.0 (7.0)
Admission creatinine		1.0 (0.43)	0.9 (0.4)	0.294	0.98 (0.4)
Admission hematocrit		39.63 (± 5.07)	40.12 (± 5.49)	0.528	39.89 (± 5.29)
Admission hemoglobin		12.83 (± 1.75)	13.01 (± 1.97)	0.519	12.93 (± 1.86)
Admission WBC count		8.02 (3.37)	7.9 (4.02)	0.721	8.01 (3.55)
Admission platelet count (×1000)		219.0 (100.0)	232.0 (104.2)	0.039	225.5 (97.0)
Admission platelet count (×1000) to WBC count ratio		28.45 (13.12)	28.97 (12.6)	0.073	28.78 (12.42)
Admission NIHSS		17.0 (7.0)	9.0 (12.0)	<0.001	14.0 (11.0)
Door to CT (minutes)		25.0 (26.0)	26.0 (28.0)	0.214	25.0 (28.25)
Baseline non-contrast CT ASPECTS on 5-mm cuts		9.0 (2.0)	10.0 (2.0)	0.603	9.0 (2.0)
Hyperdense MCA present on thin cuts?	No	31 (34.8%)	52 (52.5%)	0.022	83 (44.2%)
	Yes	58 (65.2%)	47 (47.5%)		105 (55.8%)
Stroke etiology (TOAST criteria)	Cardioembolism	50 (56.2%)	46 (46.5%)	0.2	96 (51.1%)
	Large artery atherosclerosis	11 (12.4%)	24 (24.2%)		35 (18.6%)
	Stroke of undetermined etiology	21 (23.6%)	23 (23.2%)		44 (23.4%)
	Stroke of other determined etiology	7 (7.9%)	6 (6.1%)		13 (6.9%)
Laterality	Left	44 (49.4%)	50 (50.5%)	0.622	94 (50.0%)
	Right	45 (50.6%)	48 (48.5%)		93 (49.5%)
	Bilateral	0 (0.0%)	1 (1.0%)		1 (0.5%)
Occluded segment	M1	44 (49.4%)	52 (52.5%)	0.833	96 (51.1%)
	M2	20 (22.5%)	23 (23.2%)		43 (22.9%)
	M1 and M2	25 (28.1%)	24 (24.2%)		49 (26.1%)
Hemorrhagic transformation?	No	54 (60.7%)	69 (69.7%)	0.252	123 (65.4%)
	Yes	35 (39.3%)	30 (30.3%)		65 (34.6%)
Type of hemorrhagic transformation	None	54 (60.7%)	69 (69.7%)	0.213	123 (65.4%)
	HI1	3 (3.4%)	4 (4.0%)		7 (3.7%)
	HI2	14 (15.7%)	18 (18.2%)		32 (17.0%)
	PH1	14 (15.7%)	6 (6.1%)		20 (10.6%)
	PH2	4 (4.5%)	2 (2.0%)		6 (3.2%)
Calculated rCBF 20% volume (mL)		0.0 (7.0)	0.0 (8.3)	0.479	0.0 (8.0)
Calculated rCBF 30% volume (mL)		9.2 (25.0)	8.0 (25.3)	0.911	8.6 (25.0)
Calculated rCBF 34% volume (mL)		18.0 (35.4)	13.8 (31.4)	0.684	14.7 (33.25)
Calculated rCBF 38% volume (mL)		23.4 (40.0)	20.6 (32.5)	0.53	21.5 (38.1)
Calculated Tmax 4 s volume (mL)		206.8 (105.4)	193.0 (130.1)	0.212	197.4 (129.2)
Calculated Tmax 6 s volume (mL)		116.49 (± 58.85)	90.0 (75.5)	0.028	102.5 (82.25)
Calculated Tmax 8 s volume (mL)		75.33 (± 45.75)	51.0 (62.4)	0.013	62.1 (66.0)
Calculated Tmax 10 s volume (mL)		45.0 (57.8)	33.8 (49.0)	0.047	39.2 (57.4)
CBV 34% (mL)		3.0 (15.0)	3.0 (16.5)	0.787	3.0 (15.25)
CBV 38% (mL)		5.0 (17.0)	5.0 (18.4)	0.897	5.0 (18.0)
CBV 42% (mL)		5.4 (19.4)	6.0 (21.0)	0.939	6.0 (21.0)
Calculated mismatch (mL)		94.0 (65.0)	78.0 (79.5)	0.016	83.8 (66.25)
Calculated mismatch ratio		16.8 (35.9)	18.14 (35.21)	0.992	17.84 (35.72)
Hypoperfusion index		0.4 (0.4)	0.3 (0.2)	0.035	0.33 (0.3)
Single phase CTA collateral score		2.0 (1.0)	2.0 (1.0)	0.749	2.0 (1.0)
DSA collaterals (ASITN/SIR criteria)		2.0 (1.0)	3.0 (1.5)	0.039	2.0 (1.05)
Clot burden score		6.0 (3.0)	6.2 (2.0)	0.383	6.0 (2.85)
IV TPA administered?	No	58 (65.2%)	73 (73.7%)	0.264	131 (69.7%)
	Yes	31 (34.8%)	26 (26.3%)		57 (30.3%)
Door to needle time (minutes)		1113.0 (1050.0)	1113.0 (946.0)	0.144	1113.0 (1031.0)
MT attempted?	No	6 (6.7%)	35 (35.4%)	<0.001	41 (21.8%)
	Yes	83 (93.3%)	64 (64.6%)		147 (78.2%)
Type of anesthesia	General	76 (85.4%)	59 (59.6%)	<0.001	135 (71.8%)
	Monitored anesthesia care	7 (7.9%)	5 (5.0%)		12 (6.4%)
	MT not attempted	6 (6.7%)	35 (35.4%)		41 (21.8%)
Door to groin puncture (minutes)		139.0 (88.0)	236.0 (1399.0)	<0.001	174.0 (346.25)
Groin puncture to recanalization (minutes)		32.0 (31.0)	60.0 (155.0)	<0.001	39.5 (73.35)
Door to recanalization (minutes)		180.0 (120.0)	292.0 (1429.5)	<0.001	209.0 (367.5)
Number of Passes	1	62 (69.7%)	37 (37.4%)	<0.001	99 (52.7%)
	2	14 (15.7%)	11 (11.1%)		25 (13.3%)
	3	5 (5.6%)	7 (7.1%)		12 (6.4%)
	4	1 (1.1%)	6 (6.1%)		7 (3.7%)
	5	1 (1.1%)	3 (3.0%)		4 (2.1%)
	MT not attempted	6 (6.7%)	35 (35.4%)		41 (21.8%)
mTICI	0	0 (0.0%)	6 (6.1%)	<0.001	6 (3.2%)
	1	2 (2.2%)	2 (2.0%)		4 (2.1%)
	2a	2 (2.2%)	5 (5.0%)		7 (3.7%)
	2b	21 (23.6%)	14 (14.1%)		35 (18.6%)
	2c	9 (10.1%)	7 (7.1%)		16 (8.5%)
	3	49 (55.1%)	30 (30.3%)		79 (42.0%)
	MT not attempted	6 (6.7%)	35 (35.4%)		41 (21.8%)
Discharge sodiuma		139.3 (± 2.8)	139.0 (4.0)	0.841	139.0 (4.0)
Discharge potassium ^a^		4.14 (± 0.39)	4.26 (± 0.42)	0.048	4.2 (± 0.41)
Discharge glucose ^a^		103.0 (25.0)	107.0 (29.5)	0.155	104.1 (25.25)
Discharge calcium ^a^		8.77 (± 0.54)	8.9 (0.7)	0.092	8.86 (0.7)
Discharge BUN ^a^		15.6 (7.0)	16.0 (9.5)	0.262	15.8 (8.0)
Discharge creatinine ^a^		0.9 (0.45)	0.84 (0.4)	0.318	0.9 (0.4)
Discharge hematocrit ^a^		35.16 (± 5.63)	35.62 (± 6.0)	0.592	35.4 (± 5.82)
Discharge hemoglobin ^a^		11.31 (± 1.97)	11.47 (± 2.1)	0.59	11.4 (± 2.04)
Discharge WBC count ^a^		7.91 (2.85)	8.02 (3.48)	0.978	8.01 (3.16)
Discharge platelet count (×1000) ^a^		228.0 (115.0)	254.0 (137.5)	0.055	239.0 (126.75)
Discharge platelet count (×1000) to WBC count ratio ^a^		29.14 (13.4)	31.92 (17.84)	0.08	31.06 (16.11)
Discharge BMI ^a^		26.6 (7.99)	29.15 (± 7.61)	0.112	27.1 (9.33)
Discharge HR ^a^		76.0 (27.0)	76.0 (20.5)	0.872	76.0 (25.0)
Discharge SBP ^a^		127.22 (± 19.32)	131.0 (34.5)	0.044	128.5 (27.25)
Discharge DBP ^a^		68.0 (17.0)	73.01 (± 13.4)	0.092	70.0 (17.5)
Discharge RR ^a^		18.0 (2.0)	18.0 (2.0)	0.127	18.0 (2.0)
Discharge SpO_2_^a^		97.0 (3.0)	98.0 (3.0)	0.647	97.0 (3.0)
Discharge NIHSS ^a^		2.0 (6.0)	7.0 (13.0)	<0.001	4.0 (9.25)
NIHSS shift ^a^		11.0 (5.0)	3.0 (5.0)	<0.001	7.04 (± 7.12)
90-day mRS ^a^		2.0 (2.0)	2.2 (3.0)	0.046	2.0 (2.0)

^a^ Not included as an input in the model. Abbreviations: NIHSS: National Institutes of Health Stroke Scale; SD: Standard deviation; IQR: Interquartile range; JHU: Johns Hopkins Hospitals; TIA: Transient ischemic attack; BMI: Body mass index; HR: Heart rate; SBP: Systolic blood pressure; DBP: Diastolic blood pressure; RR: Respiratory rate; SI: Shock index; BUN: Blood urea nitrogen; WBC: White blood cell; CT: Computed tomography; ASPECTS: Alberta Stroke Program Early CT Score; MCA: Middle cerebral artery; HI: Hemorrhagic infarction; PH: Parenchymal hematoma; rCBF: Relative cerebral blood flow; Tmax: Time to the maximum of the residue function; CBV: Cerebral blood volume; CTA: Computed tomography angiography; DSA: Digital subtraction angiography; ASITN/SIR: The American Society of Interventional and Therapeutic Neuroradiology/Society of Interventional Radiology; IV: Intravenous; tPA: Tissue plasminogen activator; MT: Mechanical thrombectomy; mTICI: Modified treatment in cerebral ischemia; mRS: Modified Rankin Score.

## Data Availability

The data presented in this study are available on request from the corresponding author. The data are not publicly available due to ethical restrictions and legal constraints.

## Supplementary Materials

The following supporting information can be downloaded at: https://www.mdpi.com/article/10.3390/jcm12030839/s1. Figure S1: Sorted feature importance and their mean SHAP values for the model predicting the outcome mRS at 90 days with (A) the XGBoost algorithm, (B) the CatBoost algorithm, and (C) the Random Forest algorithm; Figure S2: Sorted feature importance and their mean SHAP values for the model predicting the outcome NIHSS shift with (A) the XGBoost algorithm, (B) the LightGBM algorithm, and (C) the CatBoost algorithm.
