# Supplementary material for: Prediction of Functional Outcome in Stroke Patients with Proximal Middle Cerebral Artery Occlusions Using Machine Learning Models
Source: J Clin Med. 2023 Jan 20;12(3):839. doi: 10.3390/jcm12030839 (PMC9918022; doi:10.3390/jcm12030839)

Journal of
Clinical Medicine

MDPI

Supplementary Figures

Figure S1. Sorted feature importance and their mean SHAP values for the model predicting the outcome mRS at 90 days with (A) the XGBoost algorithm, (B) the CatBoost algorithm, and (C) the Random Forest algorithm.

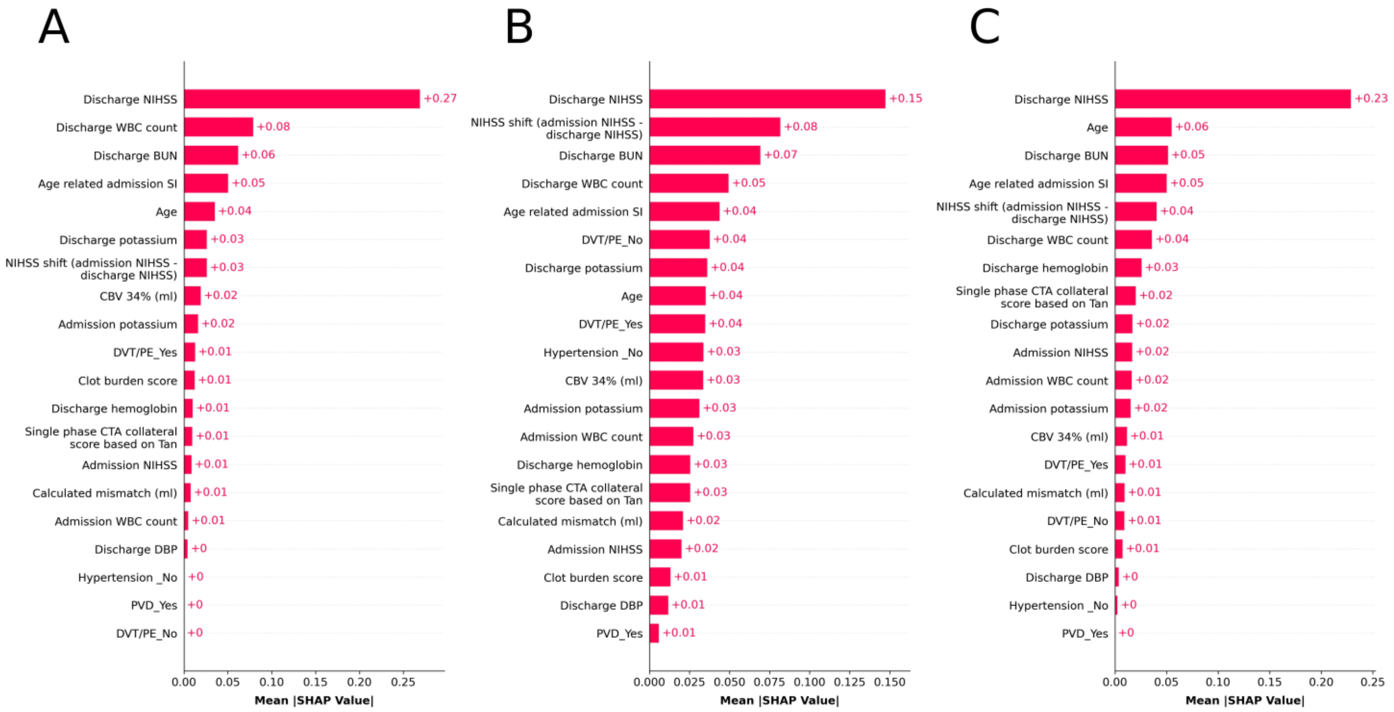

Figure S2. Sorted feature importance and their mean SHAP values for the model predicting the outcome NIHSS shift with (A) the XGBoost algorithm, (B) the LightGBM algorithm, and (C) the CatBoost algorithm.

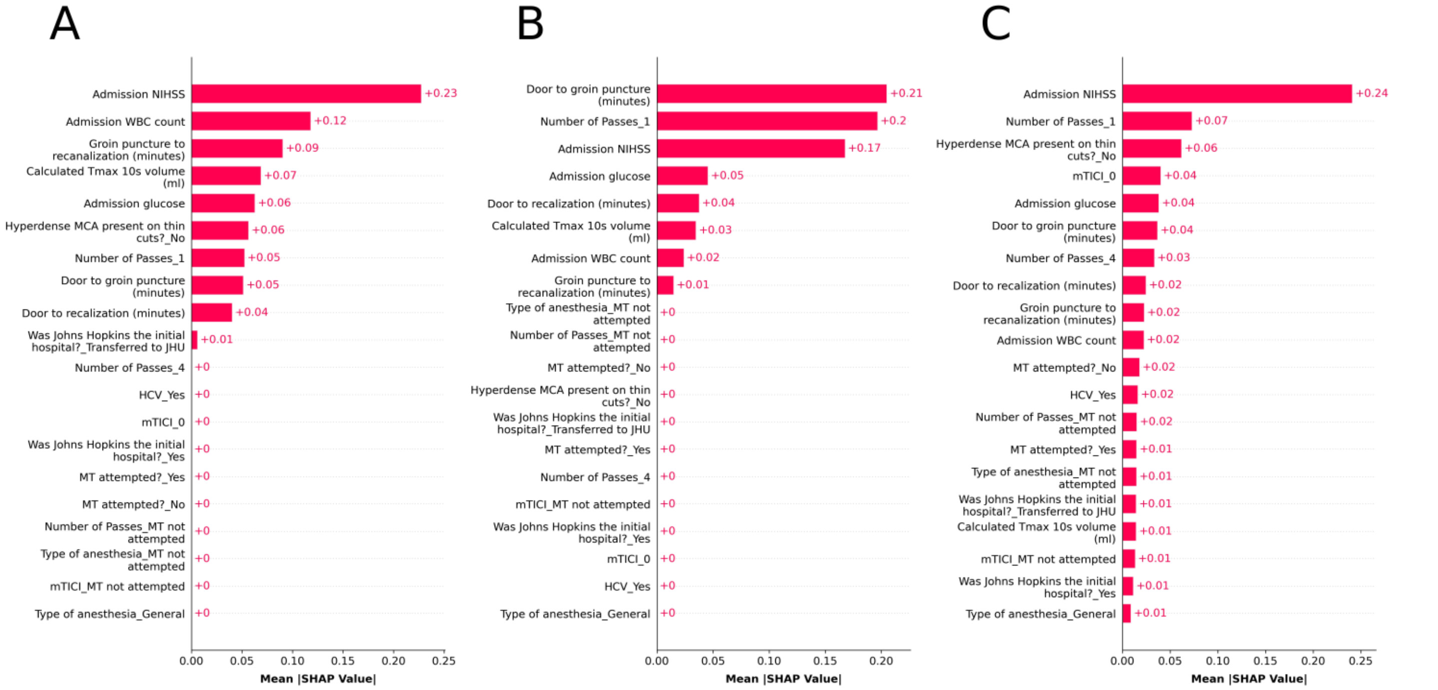

Supplement: Supplementary file 1 [file jcm-12-00839-s001.zip › jcm-2170751-supplementary.pdf]
